# From pressure to force: a device-independent, predictive model of plantar loading for personalized orthoses

**DOI:** 10.3389/fbioe.2026.1950679

**Published:** 2026-10-19

**Authors:** Sergio Fuentes del Toro, Josue Aranda-Ruíz

**Affiliations:** 1 Mechanical Engineering Department, Universidad Carlos III de Madrid, Madrid, Spain; 2 Department of Continuum Mechanics and Structural Analysis, Universidad Carlos III de Madrid, Madrid, Spain

**Keywords:** device independence, foot orthosis, gait biomechanics, ground reaction force, insole design, plantar pressure, predictive model

## Abstract

**Introduction:**

Plantar loading is conventionally characterized by peak pressure, yet peak pressure is a local force-to-area ratio set partly by the measuring device and by the foot-support interface, which limits its use as a portable input for foot-loading assessment and orthosis design. We tested whether the applied regional force is a more stable descriptor.

**Methods:**

We used two large, open-access datasets acquired on different platforms (CAD WALK, RSscan; and StepUP-P150, Stepscan). Force was calibrated on both systems through a Newton impulse identity, and every footstep was registered onto a common, length-normalized anatomical frame.

**Results:**

The body-weight-normalized applied force was more reproducible between footsteps than the peak pressure, was preserved when the interface changed from barefoot to shod (a non-significant 0.7% change against a 32.5% collapse in peak pressure over the same trials), and was captured by a closed-form power law of body mass and walking speed that reproduced the measured force with a coefficient of determination of 0.96 (0.955 under leave-one-subject-out cross-validation), whereas the same model form explained only 0.33 of the peak pressure. On the common frame, the two platforms agreed on a single per-cell load map (cross-system correlation 0.95).

**Discussion:**

We release a device-independent loading specification, comprising barefoot regional force targets (0.86 body weight at the rearfoot and 1.10 at the forefoot), the universal load map, and the fitted model, that reconstructs the plantar force field of an individual from the body mass, walking speed, and foot length as a direct input for the design of personalized foot orthoses.

## 1 Introduction

Plantar pressure measurement is the standard way to quantify how the foot loads the ground. Whether it is recorded on an instrumented walkway or with an in-shoe system, the quantity most often reported, and the one most often used to assess feet and specify footwear and orthoses, is the pressure peak, which is taken either over the whole foot or within anatomical regions (Cavanagh and Lafortune, 1980; Fourchet et al., 2012; Horiguchi et al., 2025; Tessutti et al., 2010). Peak pressure is intuitive, it is returned directly by the sensor, and it maps naturally onto tissue-level concerns such as focal overload and ulceration, so it has become the *de facto* design input for load-bearing foot devices.

This choice has become consequential as foot orthoses move from uniform inserts toward personalized devices whose support is tailored to the individual and can differ from one region of the sole to another (Ma et al., 2024; Owings et al., 2008; Ren et al., 2024). Such a device is only as good as the loading map it is designed to meet. Yet, that map is almost always specified in peak pressure, inherited from measurement convention rather than chosen because it is the mechanically correct input. When the design target is the wrong quantity, the effort of personalizing a device is spent matching a number that the device itself changes once it is worn.

Peak pressure, in fact, is not the mechanical load the body imposes. It is the local quotient of a force and the contact area that forms at the foot-support interface, and that area is set as much by the interface, the skin, sock, midsole or insole geometry, and even the sensor resolution, as by the person. The mechanically primary quantity is the force transmitted through the foot, which is fixed by the body mass and gait dynamics through Newton’s second law and is, in principle, independent of how the contact is distributed. The distinction matters because a device is built to carry a load, not to reproduce a pressure that its own presence will alter.

Two testable consequences follow. First, because the applied force is constrained by the impulse the body must exchange with the ground on every step, regional force should be more reproducible between steps, and should stabilize in fewer steps, than peak pressure, which is free to drift with small changes in contact geometry. Second, force should scale predictably with the variables that set gait dynamics, namely, the body mass and walking speed, through the ground reaction force (Grabowski and Kram, 2008; Nilsson and Thorstensson, 1989; Yu et al., 2021), whereas peak pressure, being an interface output, need not do so.

The existing literature is consistent with this view without having tested it directly. The relationship between body weight and plantar pressure is reported as inconclusive, with some studies finding an effect confined to the midfoot and others finding none (Birtane and Tuna, 2004; Wolff et al., 2023). This absence of a clean anthropometric predictor is usually treated as measurement noise. We read it instead as a signal: if the foot accommodates added weight by recruiting more contact area, both the mean and peak pressure can remain flat while the applied force rises, which would explain the missing correlation and recast it as evidence that force, not pressure, is the conserved quantity.

Three gaps remain. First, although the reproducibility of peak pressure has been examined and the number of footsteps needed to estimate it has been questioned (Van Der Leeden et al., 2004; Wolff et al., 2023), regional force and peak pressure have not been tested side-by-side on large samples to ask which quantity a load-bearing device should be specified from. Second, the interface-invariance of force has not been tested directly; that the applied force survives a change from barefoot to shod while the pressure it produces does not is assumed rather than quantified. Third, plantar datasets are almost always analyzed within a single measurement system, so it is unknown whether a spatial loading template is a property of walking or of the measuring platform. As a result, the field lacks a reliable, predictable, and device-independent description of foot loading that can serve directly as a design input.

We close these gaps using two large, open-plantar-pressure datasets acquired on two different platforms: the CAD WALK healthy-control dataset, recorded barefoot on an RSscan platform (Booth et al., 2018), and StepUP-P150, recorded barefoot and shod at four walking speeds on a Stepscan platform (Larracy et al., 2025b). Using these data, we (i) compare the between-step reliability of regional force, peak pressure and contact area, and the number of steps each requires; (ii) show that peak pressure does not scale with body weight while force and contact area do, and we quantify the compensation; (iii) derive a scaling law for regional force in body mass and walking speed and show that force is predictable from speed while peak pressure is not; (iv) show, by comparing barefoot and shod walking in the same participants, that changing the interface leaves regional force essentially unchanged while it collapses peak pressure; and (v) show that the spatial distribution of force collapses onto a single, foot-length-scaled map that is consistent across both platforms. We release this map and the associated regional force targets as a ready, device-independent loading specification for personalized foot orthoses, a contribution aligned with reliable musculoskeletal-support technologies (United Nations Sustainable Development Goals 3 and 9).

## 2 Materials and methods

Foot loading was characterized along two parallel tracks, one per dataset, that share a common procedure and converge on a single set of quantities defined on a common spatial frame (Figure 1). The procedure is organized as an ordered sequence of steps; each step below carries the same heading used in Figure 1, and where a platform required specific handling, it is stated within the corresponding step. Symbols are defined at first use, and the methodological choices are supported by the literature as they are introduced.

**FIGURE 1:**
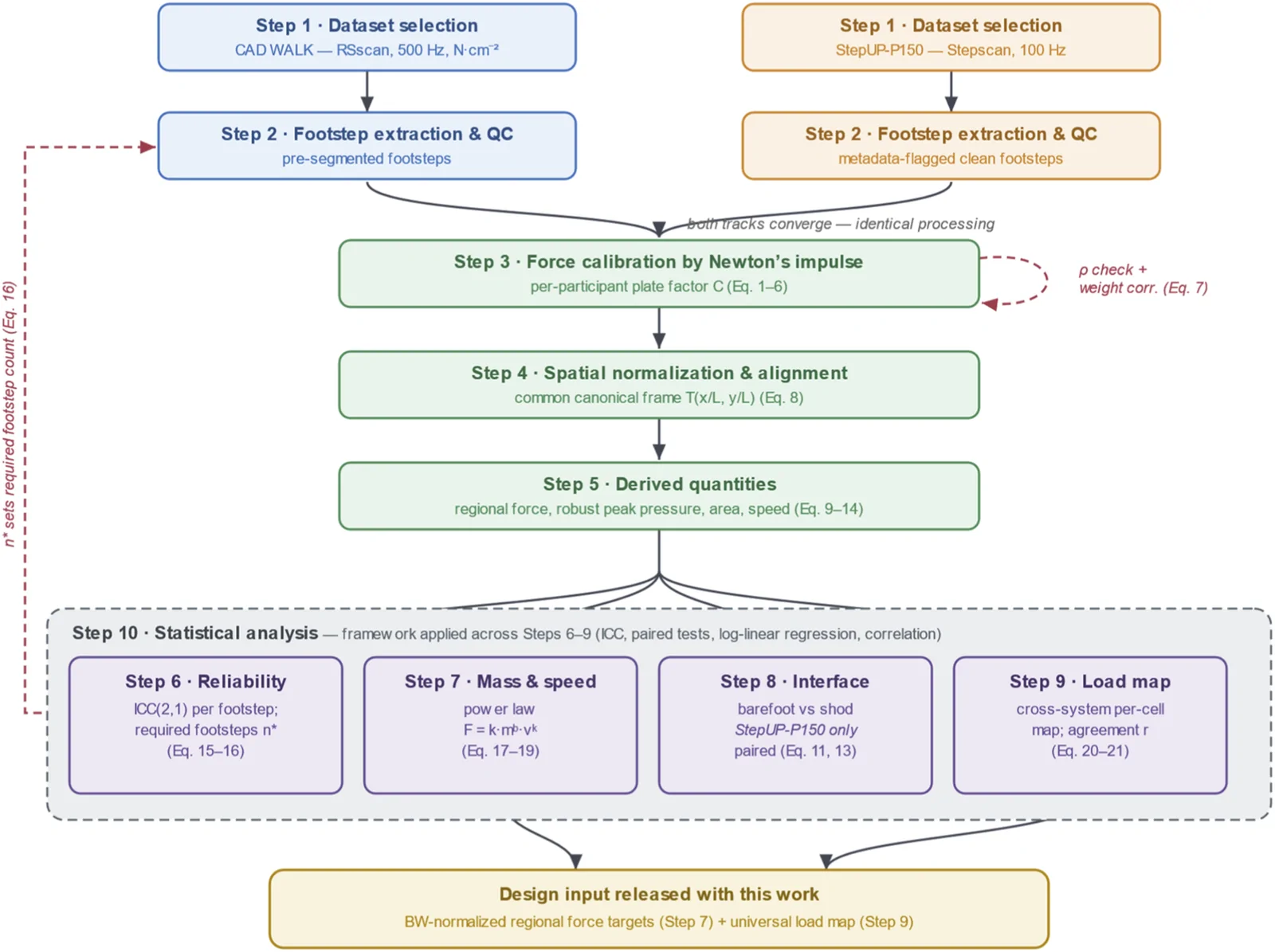
Overview of the analysis procedure. Blue and orange boxes are the two per-dataset processing tracks (CAD WALK and StepUP-P150); green boxes are the shared processing applied identically to both datasets on the common frame; purple boxes are the four parallel analyses; the dashed gray container marks the statistical framework (step 10) applied across them; the gold box is the design input released with this work. Solid arrows denote the forward data and processing flow; dashed red arrows denote feedback loops [impulse-based calibration check and weight correction (7); footstep-count requirement (16)]. Equation numbers refer to Section 2.

### 2.1 Step 1. Dataset selection

We used two large, open-access plantar-pressure datasets, both collected by their original investigators with institutional ethical approval and released in de-identified form, which removed the need for additional ethical approval for the present secondary analysis. The CAD WALK healthy-control dataset (Booth et al., 2018) was analyzed in full: all 55 adults of the healthy-control cohort (2,640 footsteps) were recorded barefoot at a self-selected walking speed (1.34 ± 0.14 m s^-1^, range 1.10–1.68) on an RSscan footscan platform with rectangular 7.62 mm by 5.08 mm sensors sampled at 500 Hz, with pressure expressed in Ncm^-2^; each trial file contains a single, pre-segmented footstep. From StepUP-P150 (Larracy et al., 2025b), we analyzed all the participants recorded (55) on a Stepscan platform with square 5 mm by 5 mm sensors sampled at 100 Hz, under two interface conditions (barefoot and a standardized sneaker) and four walking speeds; the preferred, slower-than-preferred, and faster-than-preferred trials were retained as steady-state walking (barefoot mean speeds 1.13 ± 0.12, 0.82 ± 0.17, and 1.43 ± 0.19 m s^-1^, respectively; overall range 0.43 m s^-1^–1.94 m s^-1^), and the slow-to-stop trial was excluded. Within each participant and condition, all valid footsteps were used. The body mass and foot length are provided in both datasets. The two datasets were chosen because they sample the same behavior (level walking) on two independent measurement systems with different sensor geometry and sampling rate, which is what allows the device-independence of the results to be tested.

### 2.2 Step 2. Footstep extraction and quality control

In CAD WALK, each trial file is a single, pre-segmented footstep and was used as provided. In StepUP-P150, individual footsteps were extracted using the per-footstep metadata supplied with the dataset (Larracy et al., 2025a): contacts flagged as outliers, incomplete, standing, or otherwise excluded were discarded, so that only clean walking footsteps entered the analysis, and the recorded bounding box located each footstep. Because the StepUP-P150 tensors are temporally normalized, the true stance duration of each footstep was recovered from its recorded start and end frames. Each footstep carried a manually verified left or right label.

After quality control, 27,385 of the 30,424 recorded StepUP-P150 footsteps (55 participants, barefoot and shod, and three steady-state speeds) were retained for analysis; 3,039 footsteps (10.0%) were excluded using the dataset’s outlier (n = 2,617) and incomplete-contact (n = 1,809) flags (overlapping categories), with none flagged as standing. In CAD WALK, all 2,640 pre-segmented footsteps of the 55 participants were used.

### 2.3 Step 3. Force calibration by Newton’s impulse

Because the two platforms report pressure in different native units, we did not rely on the nominal units but calibrated force against the impulse-momentum principle identically for both platforms. BW was obtained from body mass using Equation 1,

BW=m g, (1)

where m is the participant body mass and g = 9.81 m^-2^ is the gravitational acceleration. At each frame *t*, the uncalibrated force ( $F_{\text{raw}}$ ) was taken as the pressure ( $P_{i}$ ) summed over the cells (*i*) in contact using Equation 2,

$$F_{\text{raw}}\left(t\right)=\sum_{i\in A\left(t\right)}P_{i}\left(t\right).$$ (2)

The force ( $F\left(t\right)$ ) was recovered through a per-trial calibration factor *C* using Equation 3,

$$F\left(t\right)=C\cdot F_{\text{raw}}\left(t\right),$$ (3)

and its vertical impulse (*J*) over the stance phase is calculated using Equation 4

$$J=\int_{\text{stance}}F\left(t\right)\;dt=C\sum_{f}F_{\text{raw}}\left(f\right)\;\Delta t,$$ (4)

where *f* indexes the stance frames and *t* is the inter-frame interval (2 m for CAD WALK and 10 m for StepUP-P150). Over a walking cycle, the mean vertical ground reaction force equals body weight, so that the vertical impulse of a single foot during stance equals the body weight times the step time (Cavanagh and Lafortune, 1980; Nilsson and Thorstensson, 1989),

$$J=BW\cdot T_{\text{step}},$$ (5)

where T_step_ is the step time, taken as the interval between successive contralateral foot contacts. Equations 4 and 5 yield the platform factor in closed form,

$$C=\frac{BW\cdot T_{\text{step}}}{\sum_{f}F_{\text{raw}}\left(f\right)\;\Delta t}.$$ (6)

The factor C (Equation 6) is computed per trial, that is, one value for each combination of participant, interface condition (barefoot or shod), and walking speed, and not one value per platform. Across the 330 StepUP-P150 trials, C had a mean of 0.0391 with a coefficient of variation of 7.8% (within-participant coefficient of variation approximately 4%), so the scaling is stable and not dominated by any single trial.

The calibration ( ρ ) (Equation 7) was verified through the ratio of the measured stance impulse to the value required by Equation 5,

$$\rho =\frac{J_{\text{meas}}}{BW\cdot T_{\text{step}}},$$ (7)

where J_meas_ is the impulse computed from the calibrated force; a value of ρ close to unity indicates internal consistency of the calibrated forces, and a small, systematic weight-dependent deviation was corrected.

### 2.4 Step 4. Spatial normalization and alignment of the plantar maps

To compare the spatial distribution of load across participants and across platforms, every footstep was brought onto a common anatomical frame, following the general strategy of registering plantar pressure images to a normalized foot template (Booth et al., 2020; Dindorf et al., 2025). The registration was performed in three stages. First, the footprint was resampled to an isotropic 2-mm grid, which removes the difference in native resolution between the anisotropic RSscan cells and the square Stepscan cells. Second, the footprint was aligned using the temporal sequence of stance rather than its instantaneous shape, which is unstable on noisy prints: exploiting the fact that the foot rolls from heel strike to toe-off (Cavanagh and Lafortune, 1980; Horiguchi et al., 2025), the rearfoot was identified as the cells contacting earliest and the hallux as the cells contacting latest, the heel-to-hallux axis was rotated to the vertical, and the medial side was fixed from the position of the forefoot relative to that axis. Because the heel is always the first contact and the hallux the last, and the hallux lies on the medial side, this scheme is robust to the direction of travel along the walkway and resolves the left and right foot unambiguously. Third, the aligned footprint was scaled by its heel-to-hallux length onto a common canonical frame parameterized by the normalized coordinates (x/L and y/L), where L is the foot length, so that both the longitudinal and the transverse dimensions are expressed relative to the foot length and normalized to unit load,

$$\sum_{x}T\left(x\right)=1,$$ (8)

where $T\left(x\right)$ is the fraction of the footstep load carried by cell *x* of the canonical frame and the sum runs over all cells. The left and right feet were registered separately and were not mirrored onto one another. On leaving this step, footprints from both platforms occupy the same normalized, aligned frame.

### 2.5 Step 5. Derived quantities

The foot does not load as a single unit: the rearfoot, midfoot, and forefoot bear the body along the stance phase in sequence, so a loading descriptor is only meaningful when referred to a region. Each footstep was, therefore, partitioned along its length into the forefoot, midfoot, and rearfoot. We used the 40/30/30 division of foot length rather than an arbitrary split because it is the partition adopted by the plantar pressure study that provides the design targets against which our results are compared (Horiguchi et al., 2025), which keeps the regional definitions consistent across sources. This division is, therefore, an assumption adopted from the literature rather than one independently validated here; the regional force targets reported below should be interpreted as conditional on this partition, and a formal analysis of their sensitivity to the boundary placement is a direction for future work. For each region R, the peak regional force was defined as the largest value over the frames of the stance phase of the calibrated force summed over the cells of that region (Equation 9),

$$F_{R}=\max_{t}\;C\sum_{i\in R}P_{i}\left(t\right),$$ (9)

that is, the highest force the region sustains at any moment of the step, which is the load-bearing quantity relevant to design. Pressure and contact area, by contrast, were not read at each region’s own maximum but at a single common instant, the frame of peak whole-foot force, calculated using Equation 10,

$$t^{*}=\arg\underset{t}{\;\max}\;F\left(t\right),$$ (10)

where $t^{*}$ is the frame at which the whole-foot force $F\left(t\right)$ is maximal. Evaluating pressure and area at this one instant tie all the point-in-time quantities to the same biomechanically defined event of the step, so that force, pressure, and area describe the foot in a single coherent configuration rather than at three unrelated moments. A raw pressure maximum is dominated by isolated high-reading cells and by sensor spikes, and it is, therefore, not reproducible; to obtain a stable estimate, the regional peak pressure was taken as a robust upper percentile of the active-cell pressures rather than their absolute maximum, calculated using Equation 11,

$${\hat{p}}_{99}=\frac{C}{1000\;a}\;Q_{0.99}\left(\left\{\;P_{i}\left(t^{*}\right):i\in A\left(t^{*}\right)\;\right\}\right),$$ (11)

where $Q_{0.99}$ denotes the 99th percentile of the pressures of the active cells at $t^{*}$ , a is the physical area of one sensor cell, and the factor $\frac{C}{1000\;a}$ converts the calibrated reading to kPa. Taking the 99th percentile discards only the most extreme single-cell outliers while preserving the true high-load region. The regional contact area was the number of active cells at $t^{*}$ times the cell area, calculated using Equation 12.

$$A_{c}\left.=\right|A\left(t^{*}\right)\left|\;\right.a.$$ (12)

So that the regional force could be compared across participants of different body sizes, it was normalized by body weight (Equation 13),

$${\tilde{F}}_{R}=\frac{F_{R}}{BW},$$ (13)

which is also the form in which the design targets are expressed (step 7). Finally, walking speed was obtained from the forward progression of the footsteps rather than from any external timing device, as the displacement of successive footstep centroids divided by the elapsed time, taking the median over successive footsteps within a pass to be robust to the occasional mis-segmented step (Equation 14), where $c_{k}$ is the centroid of footstep k and $\tau_{k}$ is its contact time.

$$v=\underset{k}{\text{median}}\;\frac{∥c_{k+1}-c_{k}∥}{\tau_{k+1}-\tau_{k}}.$$ (14)

### 2.6 Step 6. Reliability

Between-step reliability of the regional force, the regional peak pressure, and the regional contact area was quantified with the intra-class correlation coefficient in its two-way random, absolute-agreement single-measure form, *ICC (2,1)*, selected and reported following established guidance (Koo and Li, 2016; Shrout and Fleiss, 1979),

$$ICC\left(2,1\right)=\frac{MS_{R}-MS_{E}}{MS_{R}+\left(k-1\right)\;MS_{E}+\frac{k}{n}\;\left(MS_{C}-MS_{E}\right)},$$ (15)

where $MS_{R}$ is the mean square between targets (participants), $MS_{E}$ is the residual mean square, $MS_{C}$ is the mean square between measurements, n is the number of targets, and k is the number of repeated footsteps. For each quantity, we additionally estimated the number of footsteps required to stabilize its running mean to within a fixed relative tolerance of the many-step mean (Equation 16),

$$n^{*}=\min\left\{n:\left|{\bar{x}}_{n}-{\bar{x}}_{N}\right|\leq \varepsilon \;{\bar{x}}_{N}\right\},$$ (16)

where ${\bar{x}}_{n}$ is the mean over the first n footsteps, ${\bar{x}}_{N}$ is the mean over all N footsteps, and ε is the relative tolerance and compared $n^{*}$ with the three to five footsteps common in clinical and laboratory practice (Kaufman et al., 1996; Van Der Leeden et al., 2004; Wyatt et al., 2021).

Only steady-state walking footsteps were entered into this analysis (the slow-to-stop condition was excluded *a priori*); to confirm that residual acceleration did not bias the estimate, we verified that the peak applied force did not vary systematically with footstep order within a walking pass.

### 2.7 Step 7. Scaling with body mass and walking speed

The force applied to a region of the foot is governed by two physical drivers, and the model used here follows from them rather than being imposed on the data. First, the magnitude of the ground reaction force increases with walking speed, a long-established and repeatedly replicated result (Cavanagh and Lafortune, 1980; Nilsson and Thorstensson, 1989); thus, the walking speed must enter any description of the applied force. Second, body size scales biomechanical quantities allometrically, i.e., as a power of body mass rather than in strict proportion, which is the standard framework for relating a mechanical variable to body size and the reason the mechanics of gait cannot be assumed to grow linearly with weight (Grabowski and Kram, 2008). Combining the two drivers, which act multiplicatively on the force, the natural functional form is a product of powers of the body mass and walking speed. It was fitted separately for the rearfoot ( $F_{\text{heel}}$ ) and the forefoot ( $F_{\text{fore}}$ ) because the two regions are loaded by different mechanisms during stance and need not share the same exponents,

$$F_{\text{heel}}=k_{h}\;m^{\;b_{h}}\;v^{\;\kappa_{h}},\;F_{\text{fore}}=k_{f}\;m^{\;b_{f}}\;v^{\;\kappa_{f}},$$ (17)

where, for each region, k is the scale coefficient that sets the overall force level once mass and speed are accounted for, m is the body mass, v is the walking speed, b is the mass exponent, and κ is the speed exponent. Leaving b free rather than fixing it at unity is what allows the analysis to test whether the regional force scales exactly with body weight ( b close to one) or departs from it. Taking logarithms converts the power law into a linear relation, the classical way to fit allometric laws, so that the three parameters are recovered directly by multiple linear regression rather than by non-linear fitting,

$$\ln\left(F\right)=\ln\left(k\right)+b\cdot \ln\left(m\right)+\kappa \cdot \ln\left(v\right),$$ (18)

with $\ln\left(k\right)$ , b , and κ representing the intercept and the two slopes of the regression, respectively. Because b was found to lie close to unity, expressing the regional force as a fraction of body weight absorbs the residual mass dependence and yields a size-independent quantity suitable as a universal design target (Equation 19),

$${\tilde{F}}_{\text{heel}}=\frac{F_{\text{heel}}}{BW},\;{\tilde{F}}_{\text{fore}}=\frac{F_{\text{fore}}}{BW}.$$ (19)

The speed exponent κ is the quantity of primary interest because a stable, well-determined κ is the quantitative signature that the applied force scales predictably with walking speed. The predictive value of speed was, therefore, tested in StepUP-P150 across its steady-state speeds, fitting the same power law to both the applied force and the peak pressure, so that the predictability of the two candidate loading descriptors could be compared on identical data. Beyond the regional fits, the same power law was fitted to the whole-foot total applied force over the full barefoot cohort and speed range by the same log-linear multiple regression (18); the resulting coefficients, reported in the Results, define the predictive model of the applied force that is combined with the load map in step 9. Its predictive accuracy, and that of the corresponding model fitted to the peak pressure, was quantified by the coefficient of determination between the model-predicted and the measured values, so that the applied force and the peak pressure could be compared directly as predictive inputs.

### 2.8 Step 8. Interface comparison between barefoot versus shod conditions

Using the paired barefoot and shod trials at the preferred speed in StepUP-P150, we compared the body-weight-normalized regional force (13) and the robust peak pressure (11) between the two interface conditions with paired tests, thereby isolating the effect of the foot-support interface on each quantity.

### 2.9 Step 9. Cross-system universal load map

Using the registration of step 4, a per-cell load map was obtained for each foot and each platform by averaging the unit-normalized footstep maps (8) over all valid footsteps and participants. Because each footstep map already integrates to unit load, this average is a probability-like distribution of load over the canonical frame, independent of the number of footsteps or of body size, so that maps from the two platforms are directly comparable despite their different sensor geometry and sampling rate. The two platform maps were then combined with equal weight per platform rather than by pooling all footsteps, so that the far larger footstep count of one dataset could not dominate the result and each measurement system contributed equally to the template, which is the condition consistent with the aim of a device-independent map (Equation 20),

$$T=\frac{1}{Z}\left(\frac{T_{\text{CW}}}{\sum T_{\text{CW}}}+\frac{T_{\text{SU}}}{\sum T_{\text{SU}}}\right),$$ (20)

where $T_{\text{CW}}$ and $T_{\text{SU}}$ are the CAD WALK and StepUP-P150 maps, respectively, and Z renormalizes the result to unit load. Before combining them, the extent to which the two independent systems agree is itself the test of whether a single universal map is justified, and it was quantified by the Pearson correlation ( r ) between the two platform maps over the shared cells of the canonical frame (Equation 21),

$$r=corr\;\left(T_{CW},T_{SU}\right),$$ (21)

together with a comparison of their marginal load profiles along and across the foot. A high correlation indicates that the spatial distribution of load is a property of walking rather than of the measuring device, which is what licenses averaging the two systems into one template. Finally, the scaling law of step 7 and the universal map of this step were combined into a single predictive model of the plantar load: the force carried by cell (x/L, y/L) of the canonical frame for a person of mass m walking at speed v is

$$F\left(x/L,y/L;\;m,v\right)=k\;m^{\;b}\;v^{\;\kappa }\;T\left(x/L,y/L\right),$$ (22)

where $k\;m^{\;b}\;v^{\;\kappa }$ is the total applied force from the scaling law of Equation 17, with coefficients fitted in the Results, and T is the released universal map (8). Because T sums to unity, the total force is distributed over the foot by the map, and the force of any region follows by summing T over that region. Given a participant body mass, walking speed, and foot length, the last placing the normalized map on the foot, this model predicts the force at every cell without access to either of the original platform, and together with the regional force targets of step 7, it constitutes the device-independent design input released with this work.

### 2.10 Step 10. Statistical analysis

Analyses were performed in Python with NumPy and SciPy. Reliability used the intra-class correlation coefficient (15). Paired comparisons used the paired t-test or the Wilcoxon signed-rank test according to the distribution of the differences. Scaling laws were fitted by linear regression in logarithmic form (Equation 18), and the predictive accuracy of a fitted model, including the plantar-load model of Equation 22, was reported as the coefficient of determination *R* ^2^ between the model-predicted and the measured values; associations were quantified by the Pearson or Spearman correlation coefficient. The generalization of the predictive force model (Equation 22) to unseen individuals was assessed by leave-one-subject-out cross-validation: the power law was refitted on the trials of all but one participant and used to predict the trials of the held-out participant, cycling over all 55 participants, and the cross-validated *R* ^2^ and root-mean-square error were computed from the held-out predictions. Values are reported as the mean plus or minus standard deviation, and the significance threshold was set at 0.05.

## 3 Results

### 3.1 The impulse calibration is internally consistent on both platforms

The Newton impulse calibration (step 3) recovered physically consistent forces on both measurement systems. On StepUP-P150, the plate factor *C* obtained per participant from the impulse identity (6) was stable across the cohort, with a mean of $\bar{C}$ = 0.039 and a coefficient of variation, defined as Equation 23, ${\text{CV}}_{C}$ = 7.8% (range 0.033–0.049) over the 330 combinations of participant, interface, and speed (55 participants × 2 interfaces × 3 speeds), where $C_{i}$ is the plate factor of combination i and $\bar{C}$ is the mean value over all the samples.

$${\text{CV}}_{C}=\frac{1}{\bar{C}}\sqrt{\frac{1}{N-1}\sum_{i=1}^{N}{\left(C_{i}-\bar{C}\right)}^{2}}\times 100\%.$$ (23)

Because the plate factor is a single multiplicative constant per participant, this low dispersion ( ${\text{CV}}_{C}$ ) indicates that a single, well-defined plate constant describes the platform rather than a quantity that drifts from step to step. With this calibration, the whole-foot peak force reached 1.25 times body weight on average at the preferred speed, within the physiological range for level walking and resolving the non-physical values obtained from the nominal units. On CAD WALK, whose data are already expressed in physical units, the same procedure acted as an independent check: the ratio of the measured stance impulse to the value required by body weight and step time (7) was 0.968, i.e., a systematic under-reading of approximately three per cent that increased with body mass and was corrected. The agreement of the two platforms under an identical calibration, one already in physical units and one not, is the first indication that the applied force is recovered independently of the device.

### 3.2 The applied force is more reproducible between footsteps than the peak pressure

This between-footstep reliability was assessed on StepUP-P150, whose per-footstep metadata allows the repeated footsteps of each participant to be extracted cleanly; CAD WALK, which supplies a single pre-segmented footstep per trial file, was not used here. Within participants of StepUP-P150, across repeated barefoot footsteps at preferred speed (all 55 participants), the body-weight-normalized applied force varied less from step to step than the robust peak pressure. The between-step the coefficient of variation (Equation 24), defined for each participant as the standard deviation of a quantity across that participant’s footsteps divided by its mean and then averaged over the cohort (24), was 0.18 for the whole-foot force and 0.20 for the forefoot force against 0.26 for the peak pressure (Figure 2), where $x_{pj}$ is the value of the quantity at footstep j of participant p , ${\overset{-}{x}}_{p}$ and $s_{p}$ are its mean and standard deviation over that participant’s $K_{p}$ footsteps, and P = 55 is the number of participants.

$$\text{CV}=\frac{1}{P}\sum_{p=1}^{P}\frac{s_{p}}{{\overset{-}{x}}_{p}},s_{p}=\sqrt{\frac{1}{K_{p}-1}\sum_{j=1}^{K_{p}}{\left(x_{pj}-{\overset{-}{x}}_{p}\right)}^{2}}.$$ (24)

**FIGURE 2:**
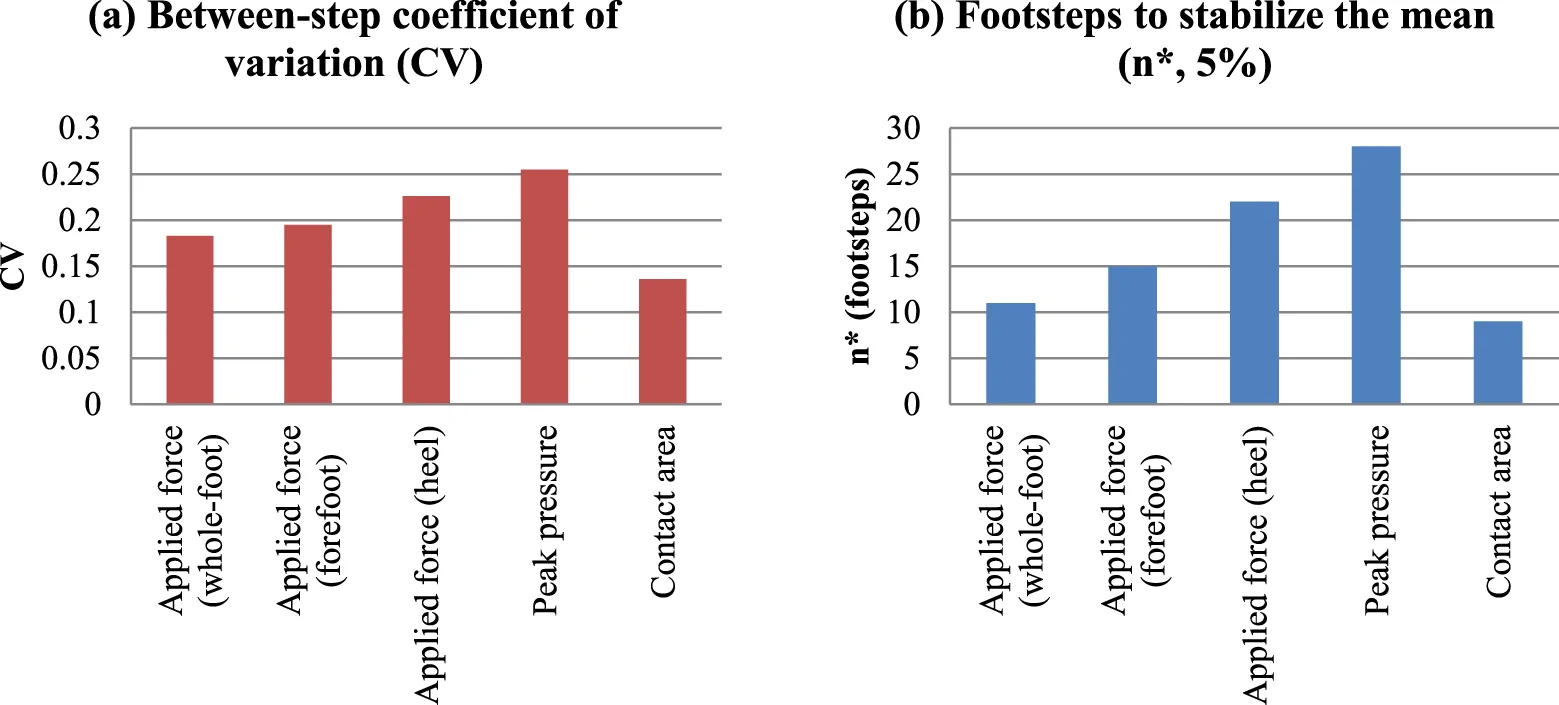
Between-footstep reliability of the candidate loading descriptors (StepUP-P150, 55 participants, and preferred barefoot speed). **(a)** Between-step coefficient of variation: the applied force (whole-foot and forefoot) varies less from step to step than the peak pressure; the contact area, a quantity that genuinely differs between participants, is shown as a reference. **(b)** Number of footsteps required to stabilize the running mean to within five per cent (n*, median with interquartile range); the shaded band marks the three to five footsteps common in routine practice. The applied force stabilizes in approximately half the footsteps needed by the peak pressure.

Consistently, the number of footsteps required to bring the running mean to within five per cent of its stable value (16) was 11 for the whole-foot force and 15 for the forefoot force against 28 for the peak pressure, so that the applied force stabilizes in roughly half the footsteps needed by the peak pressure, although at this strict tolerance, both exceed the three to five footsteps common in routine practice. Footstep order within a pass had no systematic effect on the peak applied force (mean within-pass regression slope −0.0023, SD 0.048; one-sample t-test p = 0.15; 881 passes), so the required footstep counts reflect genuine step-to-step variability rather than the initial acceleration. The single-measure, absolute-agreement intra-class correlation ICC (2,1) of Equation 15 is reported for completeness and behaved as expected for these quantities: it was near zero for the body-weight-normalized force (0.03 whole-foot and 0.11 forefoot), higher for the peak pressure (0.23), and highest for the contact area (0.71). This ordering does not contradict the reproducibility result but reflects a defining property of the ICC, which expresses the between-participant variance as a fraction of the total variance and, therefore, measures the ability to tell participants apart rather than the step-to-step stability of a single measurement. The body-weight-normalized force is nearly identical across healthy walkers, the very feature that makes it a universal design target (Section 3.6), so its between-participant variance, and hence its ICC, is small by construction; the contact area, which differs markedly between large and small feet, yields a high ICC and serves as a positive control that the estimator is sound. Absolute step-to-step reproducibility, which governs how many footsteps a reliable measurement requires, is captured by the coefficient of variation and by the stabilization count, and both favor the applied force over the peak pressure.

### 3.3 The applied force is invariant to the foot-support interface, whereas the peak pressure collapses

The comparison between the paired barefoot and shod trials at preferred speed (Step 8), on all 55 participants, produced the central dissociation of this work (Figure 3). The body-weight-normalized applied force was preserved when the interface changed from barefoot to shod, moving from 1.251 ± 0.060 to 1.260 ± 0.076 times body weight, a change of 0.7% that was not statistically significant (paired t-test, p = 0.26). Over the same trials the robust peak pressure fell from 572 ± 79 to 386 ± 50 kPa, a reduction of 32.5% that was highly significant (paired t-test, p < 10^–25^). Taken per participant, the mean absolute change induced by the interface was 3.7% for the applied force and 31.8% for the peak pressure, so that the peak pressure was 8.6 times more sensitive to the interface than the force. The two descriptors therefore separate cleanly: inserting a shoe between the foot and the plate leaves the force transmitted through each region essentially unchanged while it redistributes and cushions the pressure, which is precisely the quantity that a pressure-based design would read as a large effect. The applied force, being the mechanically conserved quantity, is unaffected.

**FIGURE 3:**
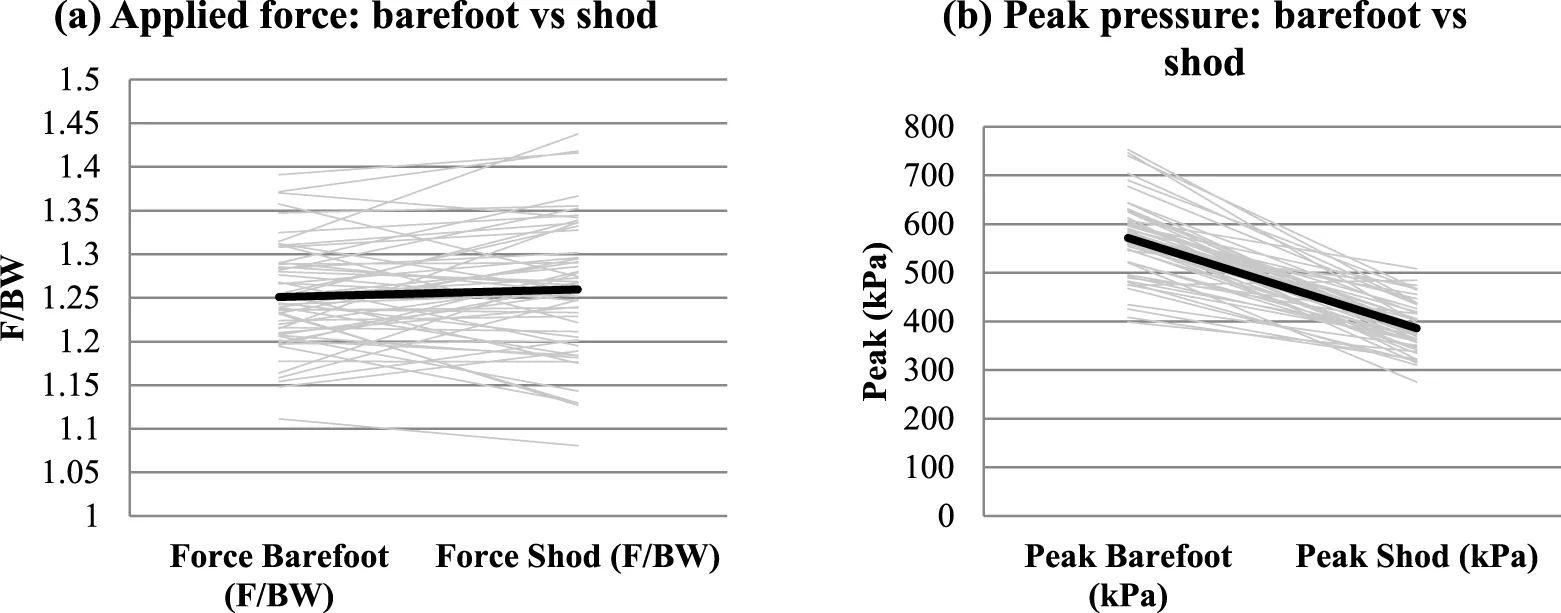
Dissociation of the applied force and the peak pressure under a change of foot-support interface (StepUP-P150, 55 participants, preferred speed). Each thin line joins the barefoot and shod value of one participant; the bold line is the group mean. **(a)** The body-weight-normalized applied force is preserved from barefoot to shod (1.251–1.260 BW, +0.7%, not significant). **(b)** The robust peak pressure collapses (572–386 kPa, −32.5%, p < 10^–25^). The interface leaves the transmitted force essentially unchanged while it redistributes and cushions the pressure.

### 3.4 The applied force scales predictably with walking speed

Across the steady-state speeds of StepUP-P150, spanning 0.43 m s^−1^–1.94 m s^−1^ in the barefoot condition (165 trials), the applied force followed the power law of Equation 17 (Figure 4) with a body-weight-normalized form F/BW = 1.243 v^0.179^, explaining half of the variance (*R* ^2^ = 0.51, r = 0.76, and p < 10^–26^). The peak pressure also increased with speed, as peak = 548 v^0.30^ (*R* ^2^ = 0.30, r = 0.51, and p < 10^–13^), but with markedly more scatter: at any given speed, the peak pressure was far less determined than the applied force. The applied force is thus not only reproducible and interface-independent but also the more predictable of the two descriptors from the primary gait variable, so that it can be estimated in advance from the body mass and walking speed, which the peak pressure cannot to the same degree.

**FIGURE 4:**
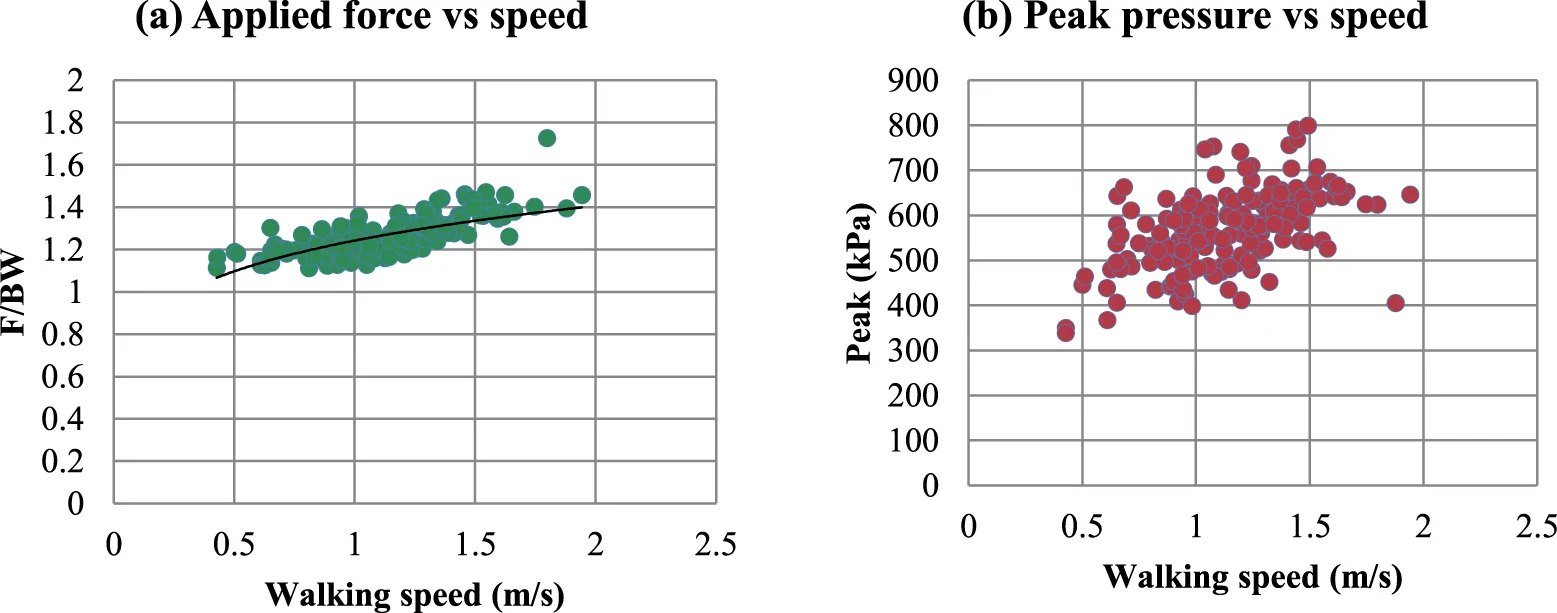
Predictability of the applied force and the peak pressure from walking speed (StepUP-P150, barefoot, 165 trials, 0.43 m s^−1^–1.94 m s^−1^). **(a)** Body-weight-normalized applied force against speed with the fitted power law F/BW = 1.243 v^0.179^ (*R* ^2^ = 0.51). **(b)** Robust peak pressure against speed, peak = 548 v^0.30^ (*R* ^2^ = 0.30). Both increase with speed, but the applied force is the more predictable of the two, with markedly less scatter about the law.

### 3.5 Regional force targets for design

Expressed in the body-weight-normalized form of Equation 19, the regional applied forces provide the loading targets released with this work. On CAD WALK the rearfoot carried 0.858 ± 0.078 and the forefoot carried 1.101 ± 0.066 times the body weight at a self-selected speed. The barefoot StepUP-P150 forces agreed in magnitude and in antero-posterior order, with the forefoot bearing more than the rearfoot, despite the different platform, sensor geometry, and sampling rate. When the interface changed to shod, the total was conserved but the distribution shifted rearward, with the rearfoot rising and the forefoot falling by a few per cent, which confirms that the interface acts on the distribution of load and not on its total and that the barefoot regional forces are the appropriate size-independent design targets.

### 3.6 A single load map describes both platforms

On the common canonical frame (step 4), the per-cell load map was highly consistent across participants, with a between-participant correlation of 0.892 ± 0.036 for the normalized map, so that the spatial distribution of load, once normalized to unit total and to foot length, is essentially universal. The map recovered the expected anatomy, a loaded rearfoot, a low-load medial arch, an oblique metatarsal ridge, and a single medial hallux, with the highest-load cells, the rearfoot and the metatarsal heads, also being the most stable across the participants and the low-load periphery being the most variable, so that the cells that matter most for design are the best determined (Figure 5). The CAD WALK and StepUP-P150 maps agreed with a cross-system Pearson correlation of r = 0.95 (0.95 for the right foot and 0.96 for the left) (21), and their marginal load profiles coincided after length normalization (*R* ^2^ = 0.70), which is what licenses averaging the two systems, with equal weight per platform (20), into the single universal load map released here (Figure 6). Together with the regional force targets of Section 3.5, this map constitutes the device-independent design input of the present work. The foot width-to-length ratio was stable across participants (mean 0.40, between-participant coefficient of variation 3.9%, and range 0.37–0.45), so the length-based normalization preserves the transverse geometry to within approximately four per cent across individuals.

**FIGURE 5:**
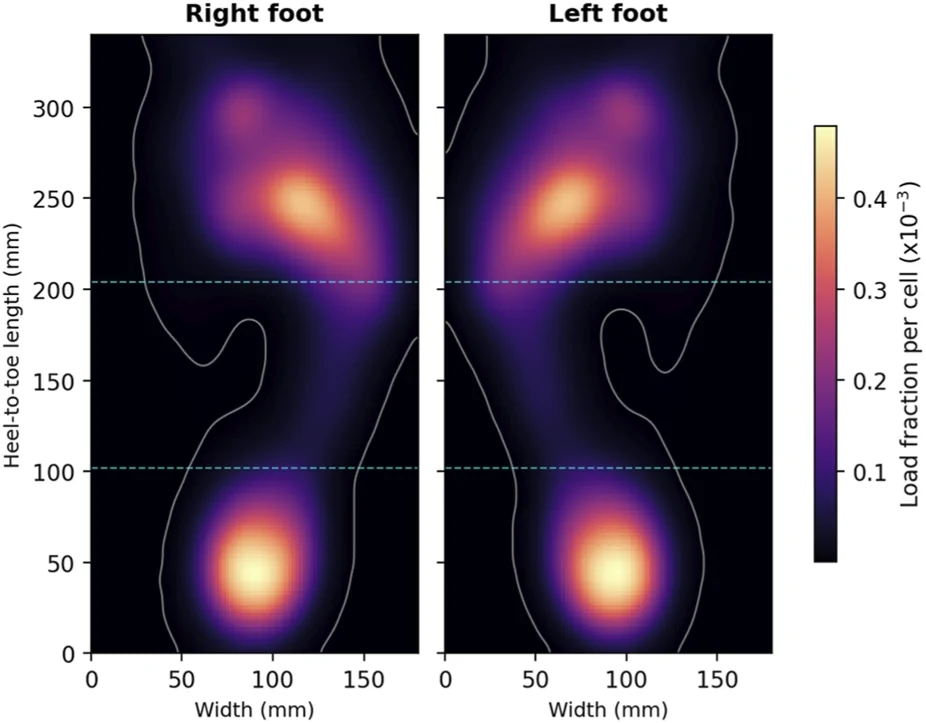
Universal per-cell load map on the common canonical frame, for the right and left foot, combining CAD WALK and StepUP-P150 with equal weight per platform (barefoot). Color encodes the fraction of stance load carried by each cell; the dashed lines mark the rearfoot, midfoot, and forefoot sections. The map recovers a loaded rearfoot, a low-load medial arch, an oblique metatarsal ridge, and a single medial hallux; the highest-load cells are also the most reproducible across participants.

**FIGURE 6:**
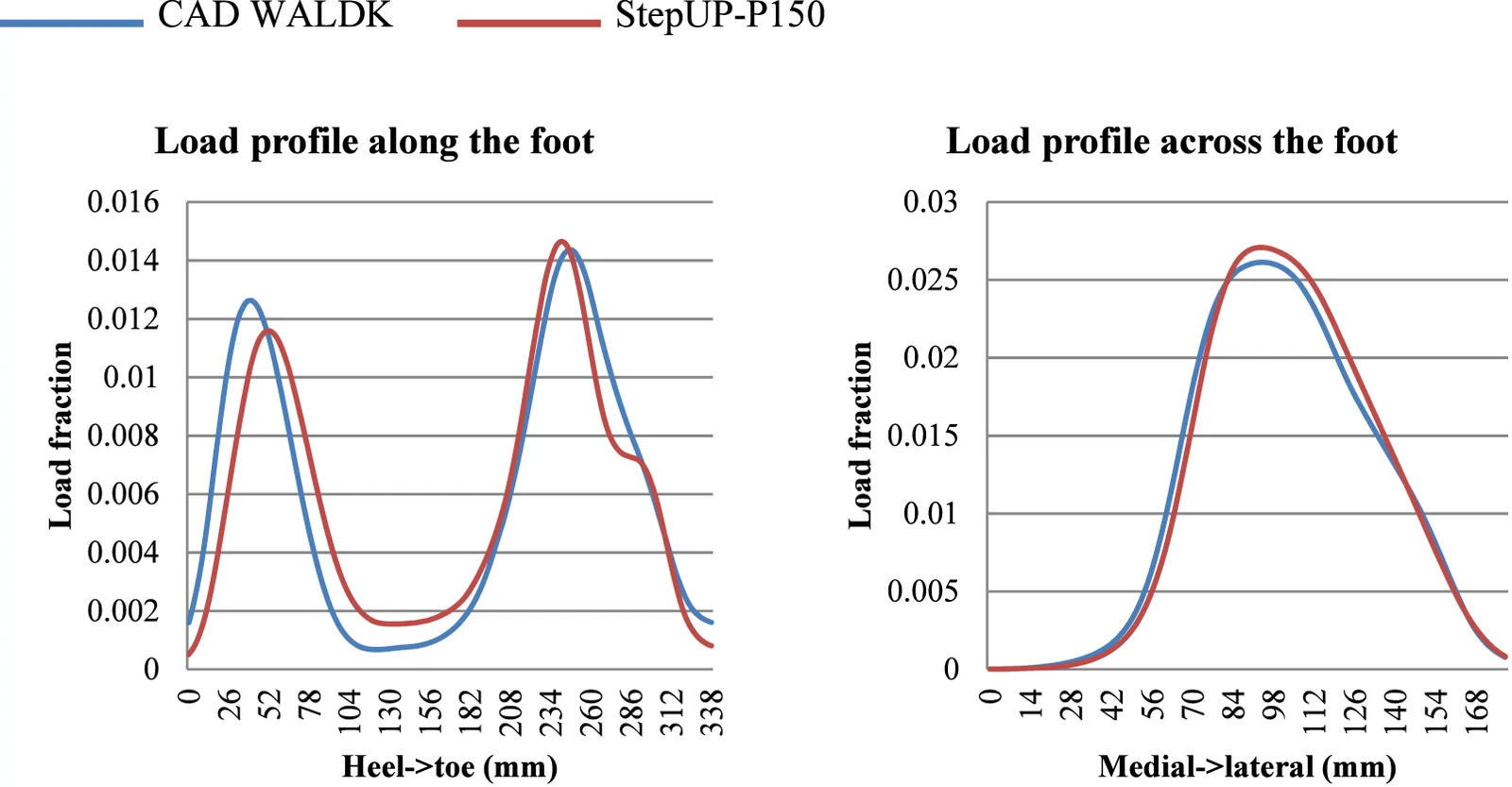
Marginal load profiles of the universal map along the foot (heel to toe) and across the foot (medial to lateral) compared between CAD WALK and StepUP-P150 after length normalization. The two independent measurement systems yield coincident profiles (*R* ^2^ = 0.70), which supports treating the combined map as device independent.

### 3.7 A predictive model of plantar loading

The scaling law and the universal map combine into a single, closed-form model that lets a reader reproduce the plantar loading of an arbitrary individual **(22)**. The total applied force follows the power law fitted across the full barefoot cohort and speed range (165 trials, 46 kg–125 kg, 0.43 m s^−1^–1.94 m s^−1^), F = k m^b^ v^κ^, with k = 17.96, a mass exponent b = 0.910 ± 0.014, and a speed exponent κ = 0.184 ± 0.012 (m in kg, v in m s^−1^, and F in N). Refitting the identical law separately to the 165 shod trials gave closely similar coefficients (b = 0.922 ± 0.020, kappa = 0.259 ± 0.016, k = 17.07, and *R* ^2^ = 0.938). In a pooled model with a barefoot-versus-shod indicator and interaction terms, the mass exponent did not differ between conditions (difference in b = 0.012, p = 0.64) and neither did the overall scale (p = 0.63); only the speed exponent was modestly larger in the shod condition (difference in kappa = 0.074, p < 0.001). The dominant mass-force scaling, which carries most of the explained variance, is thus invariant to the foot-support interface, consistent with the interface-invariance of total regional force (Section 3.3), so the fitted law transfers to shod use, where the shod coefficients reported here can be applied directly. Using these published coefficients, the model reproduced the measured total force with a coefficient of determination of *R* ^2^ = 0.96 (Figure 7a), so that body mass and walking speed together account for essentially all of the between-trial variation in the applied force. Because the coefficient of determination reflects association rather than agreement, we also quantified agreement directly: Lin’s concordance correlation coefficient was 0.98, the root-mean-square error was 48 N (5.0% of the mean force), the mean bias was −1.3 N (0.1%), and the slope of the measured-on-predicted regression was 1.02 (SE 0.017), not significantly different from unity (p = 0.31). Plotted on identical axis scales with the line of identity (Figure 7a), the residuals are symmetric about y = x, with no systematic under-prediction at high force or over-prediction at low force. The mass exponent, which is significantly below unity (p < 10^–8^), indicates a slightly sub-proportional, allometric scaling of the force with body mass. This high *R* ^2^ and the *R* ^2^ = 0.51 reported for the normalized force in Section 3.4 are consistent rather than contradictory: Section 3.4 expresses force per unit body weight, which removes the mass effect, so its *R* ^2^ = 0.51 isolates the genuine dependence on walking speed (Figure 4a), whereas the present model retains body mass, which accounts for most of the fit (mass-only *R* ^2^ = 0.917), with walking speed adding the same effect isolated in Section 3.4 (incremental *R* ^2^ = 0.048). Fitting the identical model form with the same two predictors (body mass and walking speed) to the peak pressure, by contrast, reproduced it only to *R* ^2^ = 0.33 (Figure 7b): even with mass and speed known, the peak pressure remains largely unpredictable, which quantitatively separates the two descriptors as a predictive input. To separate the contribution of each predictor, we fitted nested models. Body mass alone reproduced the applied force to *R* ^2^ = 0.917; adding walking speed raised this to *R* ^2^ = 0.965, an incremental delta-R^2^ = 0.048 with a highly significant speed exponent (κ = 0.185, partial F (1,162) = 221, and p < 0.001). Because the calibration anchors the force impulse to body weight, part of the mass dependence is expected on mechanical grounds; the speed contribution, however, is independent of the calibration, which contains no speed term. On applying the identical descriptor and model form to the peak pressure, a quantity not anchored to body weight by the calibration, body mass explained only *R* ^2^ = 0.056 of its variances, and the full model only *R* ^2^ = 0.33. This dissociation confirms that the mass-force scaling reflects loading mechanics rather than an artifact of the calibration. The force at any cell of the foot then follows by distributing the total force over the universal map, F (x/L, y/L; m, v) = k m^b^ v^κ^ T (x/L, y/L), and the force of a region by summing the map over that region. The model generalized to individuals not used in the fitting: under leave-one-subject-out cross-validation, the cross-validated coefficient of determination was *R* ^2^ = 0.955 (RMSE = 49 N, mean absolute error 3.5%, and negligible bias), essentially identical to the in-sample value, which indicates that the model does not overfit. As a worked example, for a 70 kg person walking at 1.30 m s^−1^, the model gives a total applied force of approximately 900 N, i.e., 1.31 times the body weight, which is then multiplied by the released map T to obtain the force carried by each cell. Given only a participant’s body mass, walking speed, and foot length, together with the published coefficients and the released map, the full plantar force field is, therefore, reproducible without access to either original device, which is the practical sense in which the loading input of this work is device-independent.

**FIGURE 7:**
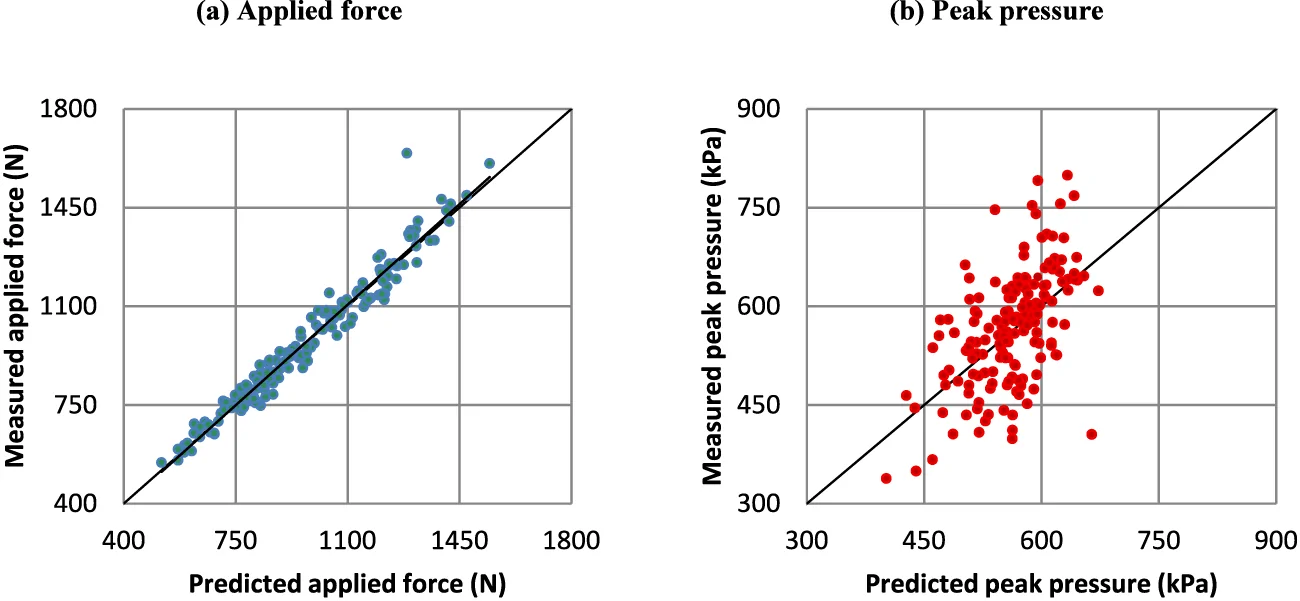
Predictive model of plantar loading (StepUP-P150, barefoot, 165 trials). Measured against model-predicted values using the published power law k m^b^ v^κ^. **(a)** Total applied force is reproduced from body mass and walking speed with *R* ^2^ = 0.96, Lin concordance correlation coefficient CCC = 0.98, RMSE = 48 N (5.0% of the mean), and a measured-on-predicted slope of 1.02 (not significantly different from 1), indicating agreement and not merely association. **(b)** Same model form applied to the peak pressure reproduces it only to *R* ^2^ = 0.33 (CCC = 0.49 and RMSE = 72 kPa). The applied force is predictable in advance; the peak pressure is not.

## 4 Discussion

In this work, we set out to identify which descriptor of plantar loading is stable enough to serve as a quantitative input for foot-loading assessment and orthosis design and to characterize it on a common frame across two independent measurement systems. The central result is a dissociation between the two candidate descriptors. The body-weight-normalized applied force was reproducible between footsteps, invariant to the foot-support interface, predictable from body mass and walking speed, and expressed on a length-normalized anatomical frame, which produced a per-cell load map consistent across participants and between the two platforms. The robust peak pressure failed on each of these counts: it varied more between steps, collapsed by approximately a third when a shoe was introduced, and, although it also rose with speed, was far less determined by it. Crucially, the applied force could be captured by a single closed-form model, force as a power law of mass and speed distributed over the universal map, which reproduces the measured loading from published coefficients alone. Taken together, these findings support the applied force, not the peak pressure, as the loading input carried forward to design and deliver that input in a device-independent and reproducible form.

The interface- and device-dependence of the peak pressure is the pivot of the argument, and it is corroborated directly by the current literature. In a five-device comparison, Chockalingam et al. (2025) found peak pressure to be poorly reproducible across systems, with intra-class correlation coefficients between 0.078 and 0.676, and concluded that data from different platforms cannot be treated as interchangeable, whereas the force-time integral was the more consistent measure, which is exactly the force-over-pressure ordering we report. Comparative technical reviews attribute this to the sensitivity of peak pressure to sensor technology, spatial resolution, and protocol, so that its absolute value is not portable between instruments (Chockalingam et al., 2025). Our within-study result is the mechanistic counterpart of that observation: interposing a shoe left the transmitted force essentially unchanged (a non-significant 0.7% difference) while it cushioned and redistributed the pressure (a 32.5% reduction), and per participant, the peak pressure was 8.6 times more sensitive to the interface than the force. The pressure that a pressure-based design would read as a large effect is, therefore, largely an artifact of the measurement interface rather than a change in the mechanical demand on the foot; because the applied force is the conserved quantity, it is the one that transfers across shoes, plates, and, by extension, the devices used to acquire it.

Beyond being reproducible and interface-independent, the applied force proved predictable in the strong sense of being captured by an explicit model. Fitted across the full barefoot cohort and speed range, the total applied force followed the allometric power law F = k m^b^ v^κ^ and reproduced the measured force with a coefficient of determination of 0.96 (0.955 under leave-one-subject-out cross-validation), whereas the same model form applied to the peak pressure reached only 0.3. This near-threefold gap in explained variance is the quantitative core of the force-over-pressure argument: mass and walking speed essentially determine the applied force but leave most of the peak pressure unexplained. We note explicitly that the impulse-based calibration anchors the calibrated force to body weight, so the strong dependence of the applied force on body mass is expected analytically and is not presented as an independent finding; it serves as a consistency check on the pipeline. The independent predictive value of the model rests on two results: walking speed, which does not enter the calibration, contributes significantly (mass-only *R* ^2^ = 0.917, mass plus speed *R* ^2^ = 0.965, incremental delta-R^2^ = 0.048, and p < 0.001), and the same calibration applied to the peak pressure does not reproduce it from mass (mass-only *R* ^2^ = 0.056), so the mass-force scaling is not a uniform artifact of the calibration but a mechanical property of loading. The result rests on well-established gait mechanics, the increase of the ground reaction force with speed documented since Cavanagh and Lafortune (1980) and Nilsson and Thorstensson (1989) and re-characterized in recent speed- and size-resolved studies (Jiang et al., 2024; Xiang et al., 2024), and on the allometric scaling of mechanical variables with body size (Grabowski and Kram, 2008); consistent with the latter, our mass exponent was significantly below unity (b = 0.910), a slightly sub-proportional scaling rather than strict proportionality. The predictability of ground-reaction quantities is itself a growing theme: the active peak and impulse of the vertical ground reaction force can be estimated from a single wearable sensor to within a few per cent (Bogaert et al., 2024), and whole-body ground reaction force and joint dynamics can be estimated from the leg length, body mass, and cadence alone (Zhao et al., 2026). Our contribution is complementary and, for design, more directly usable: rather than predicting a whole-body force trace, we predict the regional plantar force and distribute it over a device-independent per-cell map, so that the full plantar force field of an arbitrary individual is reconstructed from three inputs and a released set of coefficients. By contrast, the peak pressure, dominated by device and interface effects, is not a governable design input in this sense.

The reliability analysis requires a careful reading that ultimately reinforces this position. Between footsteps, the applied force varied less than the peak pressure and stabilized its running mean in roughly half as many steps, with both favoring the force as the more reproducible single-footstep measurement. The single-measure intra-class correlation, however, was low for the body-weight-normalized force and higher for the peak pressure and the contact area, an ordering that at first appears to contradict this. It does not: the intra-class correlation expresses between-participant variance as a fraction of total variance and, therefore, measures the ability to tell participants apart, not the stability of a single measurement (Koo and Li, 2016). The body-weight-normalized force is nearly identical across healthy walkers, the very property that makes it a universal design target, so its between-participant variance, along with it its intra-class correlation, is small by construction, whereas the contact area, which differs markedly between large and small feet, yields a high coefficient and serves as a positive control that the estimator is sound. That same universality reappears in the load map, which was consistent across participants (between-participant correlation 0.892) and, more tellingly, agreed between the two independent systems with a cross-system correlation of 0.95 and coincident marginal profiles; its highest-load cells, the rearfoot and metatarsal heads, were also the most reproducible, so the regions that matter most for design are the best determined. This coherence across subjects and devices is consistent with the reported individuality and low dimensionality of plantar pressure fields and with the statistical-modeling and normalization frameworks used to register plantar-pressure images onto a common frame (Booth et al., 2020; Dindorf et al., 2025).

The practical implication is that an orthosis or insole is better specified from a device-independent map of regional applied force than from a device-specific field of peak pressure. This comparison concerns which descriptor should be targeted, the applied force rather than the peak pressure, and is independent of whether that descriptor is obtained by direct measurement or reconstructed from the model. We do not claim that model-based estimates outperform direct subject-specific measurement: where a direct plantar-loading measurement is available, it remains the preferred input, and the role of the model and the universal map is to supply a device-independent estimate of the applied-force field when direct measurement is unavailable. The advantage of the proposed approach is, therefore, portability and independence from the acquisition device, not superior accuracy relative to subject-specific measurement. Much of the current insole-design literature optimizes against peak pressure or its redistribution (Cheng et al., 2022; Ma et al., 2024; Peng et al., 2022; Ren et al., 2024), and *in silico* prescription pipelines increasingly personalize insole geometry from such inputs (Cracknell et al., 2025; Killen et al., 2024). The present results indicate that grounding these pipelines in the applied force, expressed as a body-weight fraction and distributed over the universal map, would make the resulting prescriptions more transferable between acquisition devices and footwear conditions because the input is then defined by the conserved mechanical demand rather than by the interface through which it happened to be measured. Across individuals, the normalized map captures the population-level component of the load distribution, which is strongly shared (between-participant correlation 0.892), rather than subject-specific features such as foot width, arch morphology, or individual loading patterns; transferability at the individual level is, therefore, not claimed and remains to be established. The regional force targets, the universal load map, and the fitted predictive model released with this work are intended as exactly such an input. The universal map is offered as a portable, device-independent input for settings in which subject-specific measurement is unavailable, rather than as a substitute for it. Its use is justified by the strong agreement of the individual normalized maps (between-participant correlation 0.892) and, in particular, by the fact that the most heavily loaded cells (the rearfoot and the metatarsal heads) are also the most reproducible across participants, so that averaging preserves the load-bearing features relevant to design while attenuating only weakly-loaded, subject-specific detail. Where subject-specific data are available, a subject-matched or stratified map would capture more individual detail; the advantage of the universal map is portability, not greater accuracy, and subject stratification is a natural direction for future refinement. Combined with the predictive law and a single measurement of foot length, the universal map reconstructs a distinct absolute plantar-force field for each individual (Figure 8), so the portability of the approach does not come at the cost of individualization: the body mass, walking speed, and foot dimensions each modulate the reconstructed field in the expected direction.

**FIGURE 8:**
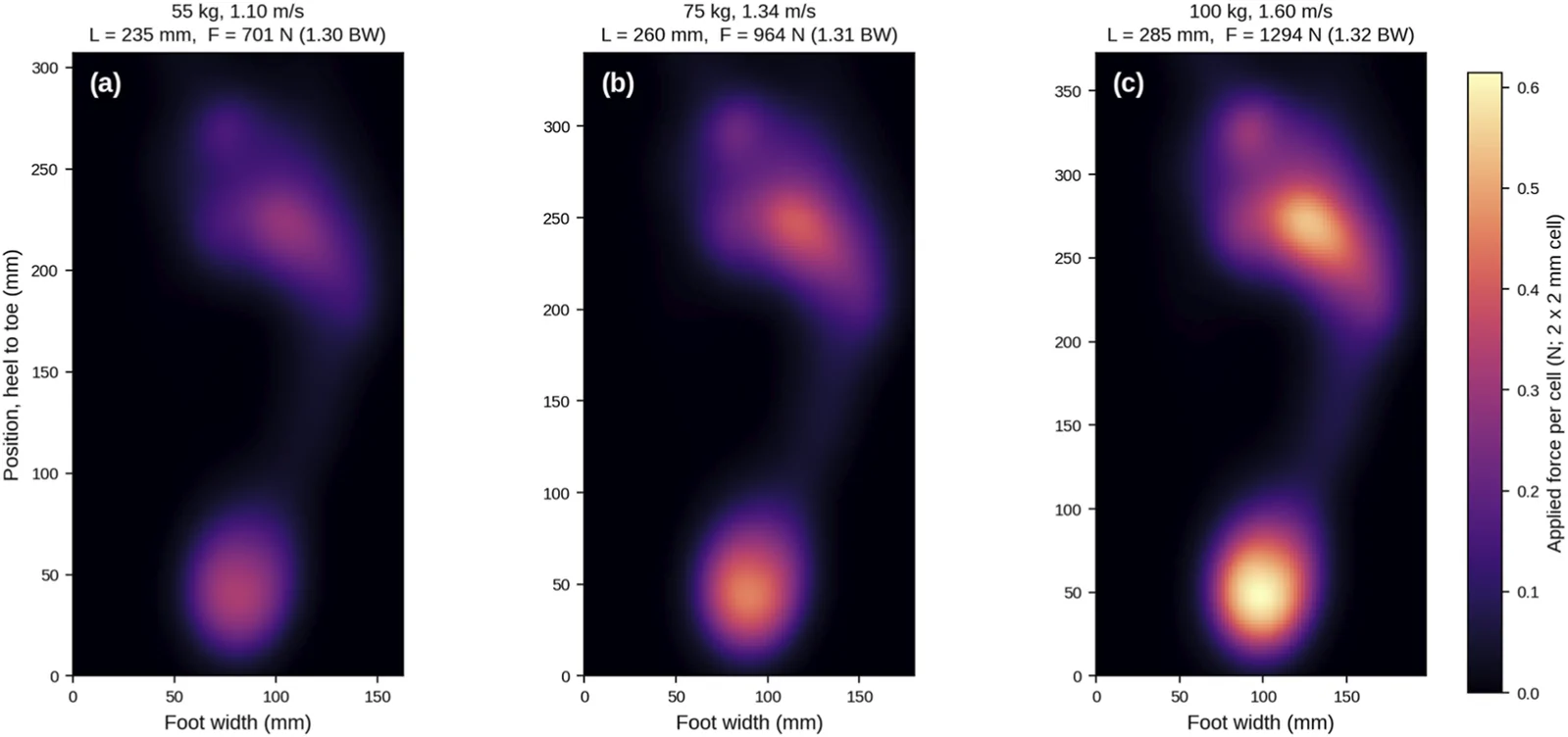
Reconstructed absolute plantar-force field for three representative individuals spanning the sample. Each field is obtained by scaling the normalized universal map to the individual’s foot length and multiplying it by the total force predicted by Equation 22. Panels: **(a)** 55 kg, 1.10 m/s, 235 mm foot, and F_total_ = 701 N; **(b)** 75 kg, 1.34 m/s, 260 mm foot, and F_total_ = 964 N; **(c)** 100 kg, 1.60 m/s, 285 mm foot, and F_total_ = 1,294 N. Color encodes the force carried by each 2 mm by 2 mm cell (N per cell) on a common scale across the three panels; axes are in millimeters. The anatomical load pattern is preserved across individuals, while the total magnitude and the footprint size scale with the body mass, walking speed, and foot length.

Figure 8 illustrates this reconstruction for three individuals spanning the range of the sample. For each individual, the normalized map is scaled to the measured foot length and multiplied by the predicted total force ( $F_{total}=k\;m^{\;b}\;v^{\;\kappa }$ ), giving the absolute force carried by each cell. The three reconstructions preserve the anatomical load pattern while differing in total magnitude and footprint size: the predicted total force rises from 701 N for the lightest, slowest individual (55 kg, 1.10 m/s, and 235 mm) to 964 N (75 kg, 1.34 m/s, and 260 mm) and 1,294 N (100 kg, 1.60 m/s, and 285 mm), and the per-cell force amplitude increases in proportion. This confirms that the framework yields a distinct, individualized absolute-force field for each person rather than a single averaged shape.

In terms of the United Nations Sustainable Development Goals, a device-independent and predictable loading input contributes to good health and wellbeing (SDG 3) by making foot-loading assessment portable across clinics and instruments and to industry, innovation, and infrastructure (SDG 9) by providing a transferable specification for personalized orthotic devices.

Several limitations bound these conclusions. The analysis was restricted to healthy adults walking at self-selected and controlled speeds on the level ground; running, stair and slope locomotion, and clinical populations such as the diabetic foot, in which the peak pressure that we de-emphasize is itself the clinical target, were not examined and are the natural next tests of generality. The predictive model was fitted and evaluated within StepUP-P150 barefoot walking, so its coefficients, and in particular the mass exponent, call for external validation on independent cohorts, and the model predicts the population-average distribution rather than an individual’s departures from it, the accuracy of which is bounded by the between-participant consistency of the map. The universal map was built from barefoot data, and the shod condition in StepUP-P150 was acquired on the platform rather than through a true in-shoe sensor, so the interface effect we report is that of the shoe as an interposed layer, not of the full in-shoe environment. Because the map is scaled by foot length in both dimensions, reconstructing an individual foot assumes an approximately constant foot width-to-length ratio; this is supported by the low between-participant variability of that ratio (coefficient of variation 3.9%), and for feet whose ratio departs most from the mean, an affine transverse rescaling by the individual width-to-length ratio would refine the reconstruction, which we leave for future work. The regional partition followed the 40/30/30 convention of the source of the design targets, and the anatomical frame rested on a temporal registration whose landmarks, although robust to walking direction and to the left and right feet, remain an approximation of foot geometry. Because this partition was adopted from the literature and not independently validated in this study, the regional force targets should be read as conditional on it; a formal sensitivity analysis of the targets to the boundary placement, performed on the per-frame force field, is left for future work. Finally, calibration and map were established on 55 participants per dataset; larger and more diverse cohorts would tighten the exponents and the map and would test whether the universality of the normalized force holds across body types beyond the ranges sampled here.

Future work follows directly. Extending the framework to running and clinical gait would test whether the applied force remains the more reliable and predictable descriptor when the loading regime and the population change, and validating the shod condition with genuine in-shoe measurement would separate the effect of the shoe as a layer from that of the complete footwear environment. Most directly, the device-independent design input assembled here, the regional force targets, the universal load map, and the predictive model, is the starting point for the mechanical design of a personalized insole, in which each region of the map is assigned a working stiffness matched to its local applied force; that design step is the subject of a companion study.

## 5 Conclusion

Across two independent plantar-pressure systems, the regional applied force, and not the peak pressure, emerged as the loading descriptor suitable for foot-loading assessment and orthosis design. The body-weight-normalized applied force was reproducible between footsteps, was preserved when the foot-support interface changed from barefoot to shod (a non-significant 0.7% change against a 32.5% collapse in the peak pressure over the same trials), and was captured by a closed-form model, a power law of body mass and walking speed, that reproduced the measured force with a coefficient of determination of 0.96 (0.955 under leave-one-subject-out cross-validation), whereas the same model form explained only 0.33 of the peak pressure. On a common length-normalized anatomical frame, the two systems agreed on a single per-cell load map, with a cross-system correlation of 0.95 and coincident marginal profiles, so the spatial distribution of load is a property of walking rather than of the measuring device. The peak pressure, by contrast, was sensitive to the interface, to the acquisition device, and, at a given walking speed, to substantial unexplained variation, which disqualifies it as a portable design input.

Beyond this comparison, the study delivers a concrete and quantitative loading specification rather than a qualitative recommendation, made of three released elements. The first is a set of barefoot regional force targets, 0.86 times the body weight at the rearfoot and 1.10 times at the forefoot, that fix the demand that each region must carry. The second is a universal per-cell load map T(x/L, y/L), defined on a common length-normalized anatomical frame and agreeing between the two independent systems at a correlation of 0.95, that fixes how that demand is distributed over the foot. The third is the fitted model F = 17.96 m^0.910^ v^0.184^, which reproduces the measured whole-foot force with a coefficient of determination of 0.96 and which, multiplied by the map, returns the force in Newtons carried by every individual cell. From three inputs alone, the body mass, walking speed, and foot length, these elements reconstruct the complete plantar force field of a person without recourse to either measuring device. The achievement is, therefore, not a description of two datasets but a portable, predictive force field that converts plantar-load characterization from a device-bound, pressure-based reading into a governable mechanical input, and it is precisely this input on which the mechanical design of a personalized insole is built in a companion study.

## Data Availability

Publicly available datasets were analyzed in this study. These data can be found here: https://www.frdr-dfdr.ca/repo/dataset/5aebad39-7977-475d-8f1b-41f22599b5a4 https://zenodo.org/records/1265420.
